# Canine filariasis in the Amazon: Species diversity and epidemiology of these emergent and neglected zoonoses

**DOI:** 10.1371/journal.pone.0200419

**Published:** 2018-07-11

**Authors:** Emanuelle Gabriela Gualberto de Argôlo, Thais Reis, Diego Ari Teixeira Fontes, Evonnildo Costa Gonçalves, Elane Guerreiro Giese, Francisco Tiago de Vasconcelos Melo, Jeannie Nascimento dos Santos, Adriano Penha Furtado

**Affiliations:** 1 Laboratório de Biologia Celular e Helmintologia, Instituto de Ciências Biológicas, Universidade Federal do Pará, Belém – Pará – Brazil; 2 Laboratório de Tecnologia Biomolecular, Instituto de Ciências Biológicas, Universidade Federal do Pará, Belém – Pará – Brazil; 3 Laboratório de Histologia e Embriologia Animal, Universidade Federal Rural da Amazônia, Instituto de Saúde e Produção Animal, Belém – Pará – Brazil; Faculty of Animal Sciences and Food Engineering, University of São Paulo, BRAZIL

## Abstract

*Dirofilaria immitis* and *Acanthocheilonema reconditum* are common parasites in dogs but have also been reported parasitizing humans. The differential diagnosis and epidemiological evaluation of these zoonoses are important to the development of efficient public health policies and control strategies. The purpose of this study was to carry out an epidemiological survey by using molecular methods for the specific identification of filarid parasites of domestic dogs in the Marajó mesoregion, State of Pará (PA), Brazil. A total of 418 canine blood samples from Marajó mesoregion (Northern Brazil) were collected, submitted to DNA extraction, polymerase chain reaction (PCR) with “pan filarial” primer, subsequent sequencing and sequence analysis using BLASTn software comparison with previously deposited sequences in GenBank. After that, a phylogenetic analysis by Maximum Parsimony was performed to aid the specific diagnosis. The obtained sequences showed the occurrence of 9 (2.15%) dogs infected with *D*. *immitis* and 30 (7.18%) by *A*. *reconditum*, with a confidence interval of 95%, there were no cases of co-infection. We observed that male dogs were more likely to *D*. *immits* and *A*. *reconditum* infection. However, age was not significant to both infections. This study reports for the first time the occurrence of *A*. *reconditum* in the northern region of Brazil and confirmed the presence of *D*. *immitis* in the Marajó mesoregion.

## Introduction

Filariasis can be caused by several onchocercid nematodes that usually have arthropods as intermediate hosts, and can be found parasitizing different species of vertebrates, as definitive hosts [1]. Canine filariases have been reported in several localities around the world, mainly in tropical regions, principally due to the greater concentration and diversity of vectors [2]. Currently, in Brazil, the following filarid species have been found to parasitize canids: *Acanthocheilonema reconditum*, *Cercoptifilaria bainae*, *Dipetalonema grassi*, *Dirofilaria immitis* and *Dirofilaria repens* [3].

In these hosts, the adult stages of *A*. *reconditum*, *C*. *bainae*, *D*. *grassi*, and *D*. *repens* are commonly found in the subcutaneous and/or intramuscular tissue and are considered to be less pathogenic [4]. On the other hand, *D*. *immitis* adults are located in the pulmonary arteries and right ventricle of the heart, and *D*. *immitis* is considered the most pathogenic of these filarid species [5].

The occurrence of these infections in Brazil is often related to *D*. *immitis* [6], and *A*. *reconditum* reports are limited to one case with a morphological description of circulating microfilariae from dogs in Minas Gerais State (Southeast) [7], epidemiological evaluations with low frequencies in Paraná State (South) [8] and Alagoas (Northeast) [9]. Until now, *A*. *reconditum* has previously not been reported previously in Brazilian Amazon, specifically in the State of Pará (North).

Zoonotic cases of *D*. *immitis* and *A*. *reconditum* occur more frequently in areas with high prevalences of infected canine hosts [4]. However, only *D*. *immitis* has been previously identified parasitizing humans in Brazil [10–12], but in The Amazon Region, which is considered endemic for canine filariasis and has climatic characteristics that favor the transmission of the parasite, only one human case has been reported [13].

Due to their higher sensitivity and specificity, molecular diagnostic methods, such as nucleotide sequence comparison, are important tools when compared with traditional methods such as blood smear analysis, immunoenzymatic and histochemical tests [14].

The specific diagnosis of these infections in domestic dogs in the Amazon is important because it can be used as a basis to direct health public policies to the proper treatment and control of this infection in both the canine and human populations [15].

Previous works reported dogs infected with *D*. *immitis* in Marajó mesoregion with high prevalence in some studied areas [16–17]. Thus, the purpose of this study was to carry out an epidemiological study using molecular tools for identification of domestic dogs infected with filarid nematodes in the Marajó mesoregion (Pará State, Brazil), expanding the analyzed area.

## Material and methods

### Sample collection

Several expeditions to Marajó mesoregion were carried out in the following municipalities: Anajás (00°59'13"S, 49°56'24"W), Chaves (00°09'36"S, 49°59'16"W), Portel (01°56′09″S, 50°49′15″W), São Sebastião da Boa Vista (01°43'04"S, 49°32'27"W) and Soure (00°43′01″S, 48°31′22″W). The expeditions occurred according to the convenience of the health authorities between the years of 2011 to 2014 and were performed by the team of the Cell Biology and Helminthology Laboratory (Institute of Biological Sciences, Federal University of Pará). A total of 418 canine blood samples from domestic dogs living in those areas were collected [Anajás (n = 70), Chaves (n = 28), Portel (n = 141), São Sebastião da Boa Vista (n = 49) and Soure (n = 130)]. The blood was collected via cephalic vein puncture, using disposable sterile needles and stored in tubes with Ethylenediamine tetraacetic acid (EDTA) in a freezer at -20°C, for molecular analysis. The blood samples were collected from dogs with respective owner’s agreement, who were informed about the purpose of this research. Blood samples were collected following the order of houses within randomly chosen neighborhoods for sampling. In houses containing more than one dog all animals were sampled except for puppies less than 3 months of age.

### Molecular diagnosis

All blood samples were submitted to DNA extraction using phenol-chloroform method [18]. The extracted DNA was subjected to PCR with DIDR F1 and DIDR R1 “pan filarial” primer, to amplify 5.8S–ITS2–28S filarid rDNA regions, according to a protocol established by the authors [14]. The amplicons were visualized after electrophoresis in 1.5% agarose gel stained with SYBR^®^ Green (*Invitrogen*, *California*, *USA*). Canine blood samples were considered positive for filariasis when one or more bands were observed.

The PCR products were cloned into vector p^®^GEM-T (*Promega*, *Wisconsin*, *USA*) which in turn was introduced into *Escherichia coli* TOP 10 electrocompetent cells (*Invitrogen*, *California*, *USA*) and sequenced with ABI 3130 model automatic DNA analyzer (*Applied Biosystems*, *California*, *USA*), in conjunction with a *BigDye*^*®*^ *Terminator v3*.*1 Cycle Sequencing Kit* (*Applied Biosystems*, *California*, *USA*), according to the manufacturer’s specifications.

For a specific diagnosis, the obtained sequences were edited and aligned in the *BioEdit* program [19]. Compared with previously deposited ones in GenBank using BLASTn program [20] additonally a phylogenetic inference was made with all the positive sequences using Maximum Parsimony (MP) method [21] in MEGA 7 program [22] to aid in the identification. The percentage of identical trees in which the associated taxa were grouped (1000 replicates) is shown next to the branches [23]. *Ascaris lumbricoides* (AB571301.1) and *Trichuris trichiura* (KC877992.1) sequences being used as an outgroups. Published sequences from *Dipetalonema reconditum*, now stated as *Acanthocheilonema reconditum* (AF217801.2), *Dipetalonema dracunculoides* (DQ018785.1), *Dirofilaria repens* (AY693808.1) and *Dirofilaria immitis* (EU182330.1) were also used for comparison with the sequences obtained in this work and for the construction of the phylogenetic tree.

All obtained sequences in this work were submitted to GenBank (accession numbers are shown in S1 Appendix).

### Epidemiological and statistical analysis

After specific diagnosis, we conducted an epidemiological analysis to investigate parameters that might influence the distribution of these infections. The prevalence of infected dogs was estimated based on specific diagnosis performed through phylogenetic inference. During sampling, information regarding age and sex of all dogs were obtained for association analysis with canine filariasis cases throughout Pearson chi-square test (X2), using the BioEstat 5.0 [24] software, with a significance level of 5% (*p*<0.05).

### Ethics statement

This study was carried out in strict accordance with the recommendations in the Guide for the Care and Use of Laboratory Animals of the National Institutes of Health. The protocol was approved by the “Conselho Nacional de Saúde, Comissão Nacional de Ética em Pesquisa, Fundação Hemopa, Ministério da Saúde” (Permit Number: 0003.0.324.000–10).

## Results and discussion

Among the 418 canine blood samples analyzed, 39 (9.33%) were considered positive for canine filariasis based in the presence of visible bands after PCR and electrophoresis. However, of the positive samples, we distinguish 9 samples with bands of approximately 500 bp and another 30 samples with bands of approximately 400 bp. None of the samples presented mixed bands; Rishniw et al. [14] also observed a similar result among their samples, and the authors suggest that this difference in amplicon size varies according to the filarid species. However, the differences observed after electrophoresis are discrete and insufficient for specific determination.

Thus, all amplicons, were subjected to nucleotide sequencing and BLASTn analysis (S1 Appendix) and 9 (2.15%) have high identity values to *D*. *immitis* sequences (between 91% to 100%), and the other 30 (7.58%) positive samples presented high identity values to *A*. *reconditum* (between 80% to 100%). Interestingly, the difference between the band patterns observed in this work was similar to that found by Rishniw et al.[14]. 500 bp for *D*. *immitis* and 400 bp *A*. *reconditum*.

**Fig 1:**
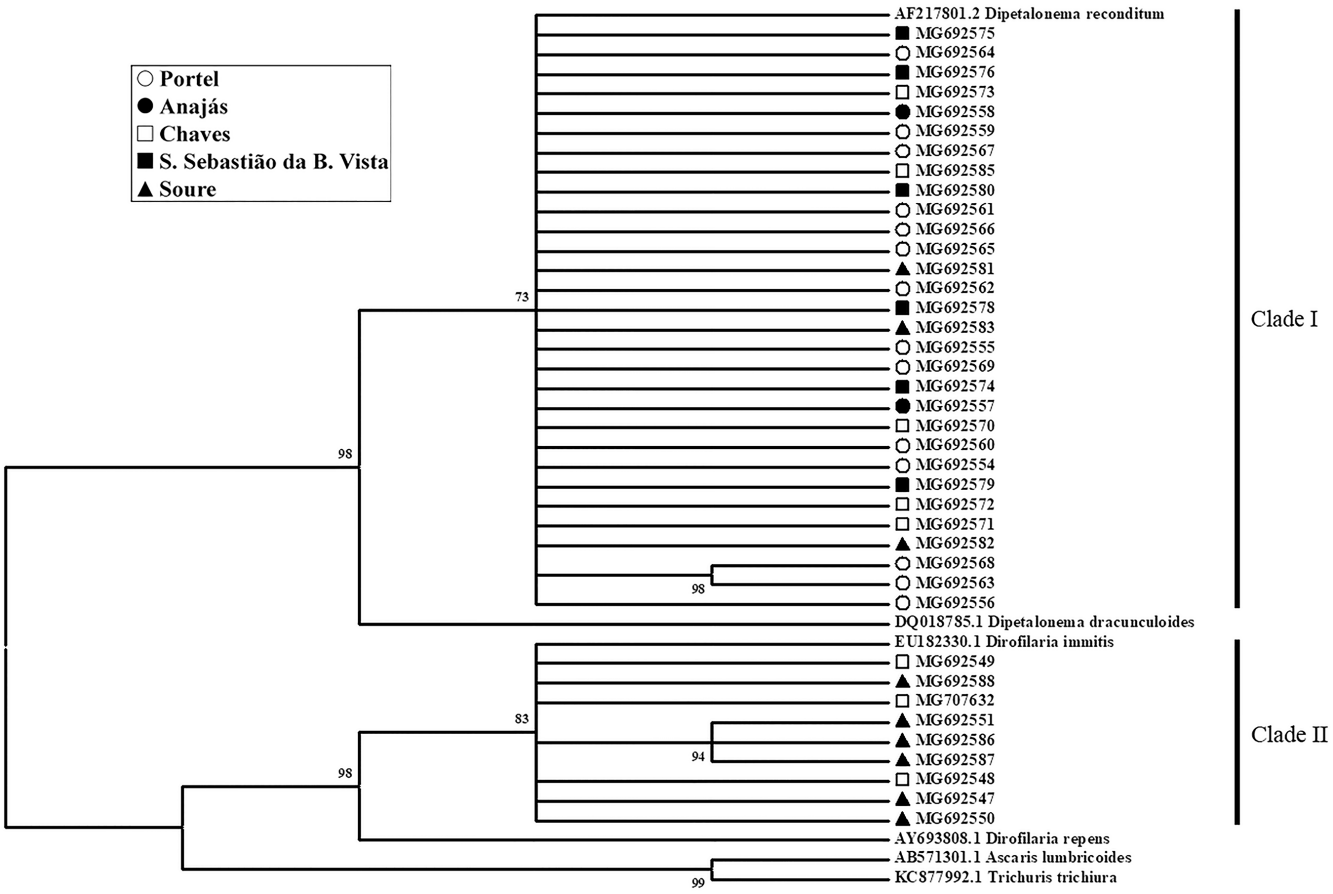
Phylogeny of filarid of the Marajó mesoregion. The cladogram was inferred using the Maximum Parsimony method. It contains a length of 266, the consistency index is 0.614458 and the retention index is 0.872170. The *Bootstrap* is indicated next to the knots of the branches. The sequence of the obtained samples were grouped in two clades: In clade I, 30 sequences of the municipalities Soure, Chaves, Portel, Anajás São Sebastião da Boa Vista formed a politomy with *A*. *reconditum* (AF217801.2), with *Bootstrap* of 73%. Clade II is composed by 9 sequences of Soure and Chaves municipalities, which form a polytomy with *D*. *immitis* (EU182330.1) withal 83% of *Bootsrap*. The other filarid sequences remained in isolated branches: *D*. *dracunculoides* (DQ018785.1) as a brother group of *A*. *reconditum* (Bootstrap 96%) and *D*. *repens* (AY693808.1) most related to *D*. *immitis* (*Bootstrap* 96%). *A*. *lumbricoides* (AB571301.1) and *T*. *trichiura* (KC877992.1) are the outgroup.

To verify the relationships between sequences and to confirm the diversity of the filarid species in dogs from the municipalities of the Marajó mesoregion, a phylogenetic analysis was carried out in all obtained sequences using MP method to generate a cladogram (Fig 1) with two distinct clades:

1. Clade I: formed by a politomy between the 30 sequences of this work and *D*. *reconditum*, now stated as *A*. *reconditum* (AF217801.2), with 73% of *Bootstrap*.
2. Clade II: formed by a politomy between the 9 sequences of this work and *D*. *immitis* (EU182330.1), with 83% of *Bootstrap*.

Furtado et al. [17] performed a filariasis diagnosis based on blood smear analysis, molecular techniques using blood samples and they considered the sequences that formed politomies in the cladogram as the species *D*. *immitis*. However these authors did not identify *A*. *reconditum* in their analysis.

This is not the first description of canine filariasis in northern Brazil. Microfilaremic dogs have been reported in several cities, example, in Belém metropolitan region, State of Pará, based on the presence of microfilariae in blood smears [25]; Salvaterra (PA), located in the Marajó mesoregion, using immunoenzymatic diagnosis methods [16]; Porto Velho, State of Rondônia [26] and Lábrea, State of Amazonas [27], using similar methods. However the epidemiological data presented in these studies were associated with *D*. *immitis* infection, but no specific method for species identification was performed. Thus, it is also possible that cases of misidentification by morphological analysis or cross-reaction in immunoenzymatic essays occurred in those studies. However, an epidemiological study in two municipalities of Marajó Archipelago (Salvaterra and São Sebastião da Boa Vista), using classical and molecular methods for diagnosis, confirmed *D*. *immitis* parasitizing dogs in these municipalities and highlighted the endemic character of this zoonosis in this area [17].

Our results show for the first time the infection of *D*. *immitis* in Anajás, São Sebastião da Boa Vista and Portel, with a lower prevalence in Soure and Chaves, additionally, *A*. *reconditum* was detected in all municipalities studied (Table 1).

**Table 1:** Distribution of filarid infection in domestic dogs by gender and age in domestic dogs from Marajó mesorregion and municipalities: Anajás, Chaves São Sebastião da Boa Vista, Portel and Soure.

	*D*. *immitis*			*A*. *reconditum*		
Municipality	Positive/tested (%)	*p*		Positive/tested (%)	*p*	
		Age	Gender		Age	Sex
Anajás	0/70 (0.0%)	-	-	2/70 (2.86%)	0.6201	0.9966
Chaves	3/28 (10.71%)	0.9248	0.0252*	5/28 (17.86%)	0.4275	0.9602
S. S. Boa Vista	0/49 (0.0%)	-	-	7/49 (14.00%)	0.4349	0.7756
Soure	6/130 (4.62%)	0.0256*	0.0675	4/130 (3.01%)	0.1960	0.9034
Portel	0/141 (0.0%)	-	-	14/141 (9.93%)	0.5784	0.0182*
Gender						
Male	9/418 (2.15%)	0.0166*		23/418 (5.45%)	0.0254*	
Female	0/418 (0.00%)			9/418 (2.13%)		
Age						
0–1 y	0/418 (0.0%)	0.0737		5/418 (1.18%)	0.2246	
>1–3 y	3/418 (0.71%)			13/418 (3.08%)		
>3 y	6/418 (1.44%)			14/418 (3.32%)		
Overall	9/418 (2.15%)			30/418 (7.58%)		

(*) significance level of 5% (p<0.05)

Previous studies in Marajó mesoregion, using molecular techniques, PCR and sequencing, have identified only *D*. *immitis* parasitizing dogs, and the prevalence described in this area differs from that found in this study, since higher percentages in Salvaterra (37.4%) and Sebastião da Boa Vista (6.67%) [17].

The dog gender showed statistically significant association for *D*. *immitis* and *A*. *reconditum* infection in Marajó mesoregion, with males having a higher probability of acquiring the infection. On the other hand, the overall age was not significant in both infections. Specifically, in Soure municipality, only age (3 or more years old) was significant for infection by *D*. *immitis*. In contrast, in Chaves, just males showed to be more susceptible to the infection. The same was observed in Portel, where males were more susceptible to *A*. *reconditum* infection.

This findings agree with studies performed by Furtado et al. [17] in the same area but differ from what has been reported for other regions [28–29]. Several authors have searched for associations between host gender or age and parasitism by filarids [30–31], and their results have been shown inconsistent, and no explanations were proposed by those associations. Similar to the results observed in the present work, we did not observe environmental or behavioral characteristics that justified the differences found in gender or age, so we infer that the cause of some associations found herein may be related to the sample number.

However the differences observed here and the factors associated with heartworm transmission in dogs need to be studied in the Amazon Region, since a complex system of interaction between domestic dogs and wild animals can provide differentiated scenarios in the dispersion of infection, as previously described [17].

The data presented in this paper describe for the first time in the Amazon Region the presence of dogs infected with *A*. *reconditum* (phylogenetic analysis). And this infection seems to be related to gender, but not to age, when analyzing the studied municipalities. None of the work reported the presence of this species of filarid in the Amazon, for this reason we cannot compare our results.

Previous studies conducted in Brazil about *A*. *reconditum* only described the occurrence, without presenting consistent epidemiological data on this infection. Unfortunately, it was not possible to evaluate if this infection is recent or has been neglected in previous studies in the Amazon, since previous work have only used morphological and immunoenzymatic techniques which may lead to an erroneous species identification. Furthermore, the distribution patterns of *D*. *immitis* also need to be minutely studied in the Amazon, because, the correct diagnosis and dynamic of infections in the dog population will be important to the development of efficient public health policies, diagnosis and control strategies of zoonotic cases.

Adult stages of *D*. *immitis* and *A*. *reconditum* are morphologically different and occupy distinct niches in the definitive host [5]. However, circulatory larval forms (microfilariae) may be confused when observed by light microscopy in routine laboratory diagnosis, even though morphological and biochemical differences have been described among them [32–34]. Although the advantages of the molecular diagnosis of canine filariasis have already been highlighted in other regions around the world [15, 17, 35–36], morphological differentiation is still necessary for taxonomic identification and the detection of *A*. *reconditum* in the Amazon Region opens up new possibilities for research on morphological and molecular differentiation of this filarid species.

Besides the diversity of canine filarid species in the extensive geographic area of the Amazon, other factors may have influence on the dynamic of those infections in the Amazon, and should be considered, as for example: (1) the high prevalence observed in previous studies; (2) the close association between humans and domestic animals cohabiting both urban and wild environments; and (3) the neglect of these infections by the public health authorities.

In the same manner that sequences of *A*. *reconditum* was described for the first time in this study, other species not yet recorded, or new species, may be detected in future studies in this area.

## Supporting information

S1 Appendix Tableau with listing of PCR positive samples and their GenBank accession numbers, as well as identities according to BLASTn and accession numbers of the corresponding GenBank sequencesb. (DOCX)
