# Supplementary material for: Canine filariasis in the Amazon: Species diversity and epidemiology of these emergent and neglected zoonoses
Source: PLoS One. 2018 Jul 11;13(7):e0200419. doi: 10.1371/journal.pone.0200419 (PMC6040735; doi:10.1371/journal.pone.0200419)
Supplement: S1 Appendix — (DOCX) [file pone.0200419.s001.docx]

| Accession | Species | Identity |
| --- | --- | --- |
| **MG692573** | *A. reconditum* (AF217801.2) | 100% |
| **MG692572** | *A. reconditum* (AF217801.2) | 82% |
| **MG692585** | *A. reconditum* (AF217801.2) | 95% |
| **MG692570** | *A. reconditum* (AF217801.2) | 89% |
| **MG692571** | *A. reconditum* (AF217801.2) | 88% |
| **MG692555** | *A. reconditum* (AF217801.2) | 91% |
| **MG692563** | *A. reconditum* (JQ039745.2) | 80% |
| **MG692567** | *A. reconditum* (AF217801.2) | 99% |
| **MG692564** | *A. reconditum* (AF217801.2) | 99% |
| **MG692559** | *A. reconditum* (AF217801.2) | 99% |
| **MG692566** | *A. reconditum* (AF217801.2) | 97% |
| **MG692561** | *A. reconditum* (AF217801.2) | 97% |
| **MG692560** | *A. reconditum* (AF217801.2) | 95% |
| **MG692554** | *A. reconditum* (AF217801.2) | 90% |
| **MG692569** | *A. reconditum* (AF217801.2) | 94% |
| **MG692562** | *A. reconditum* (AF217801.2) | 89% |
| **MG692568** | *A. reconditum* (KP420152.1) | 93% |
| **MG692565** | *A. reconditum* (AF217801.2) | 93% |
| **MG692556** | *A. reconditum* (AF217801.2) | 95% |
| **MG692558** | *A. reconditum* (AF217801.2) | 100% |
| **MG692557** | *A. reconditum* (AF217801.2) | 92% |
| **MG692579** | *A. reconditum* (AF217801.2) | 83% |
| **MG692580** | *A. reconditum* (GU593976.1) | 96% |
| **MG692578** | *A. reconditum* (AF217801.2) | 91% |
| **MG692574** | *A. reconditum* (AF217801.2) | 96% |
| **MG692576** | *A. reconditum* (AF217801.2) | 100% |
| **MG692575** | *A. reconditum* (AF217801.2) | 100% |
| **MG692581** | *A. reconditum* (AF217801.2) | 95% |
| **MG692582** | *A. reconditum* (AF217801.2) | 91% |
| **MG692583** | *A. reconditum* (GU593976.1) | 88% |
| **MG692551** | *D. immitis* (KF273905.1) | 99% |
| **MG692549** | *D. immitis* (EU182330.1) | 94% |
| **MG707632** | *D. immitis* (MF962487.1) | 100% |
| **MG692548** | *D. immitis* (KF273906.1) | 91% |
| **MG692588** | *D. immitis* (HM126608.1) | 99% |
| **MG692587** | *D. immitis* (JX866681.1) | 98% |
| **MG692586** | *D. immitis* (EU182331.1) | 99% |
| **MG692550** | *D. immitis* (KY644137.1) | 100% |
| **MG692547** | *D. immitis* (HM126608.1) | 99% |
